# Overexpression of Soluble Recombinant Human Lysyl Oxidase by Using Solubility Tags: Effects on Activity and Solubility

**DOI:** 10.1155/2016/5098985

**Published:** 2016-01-31

**Authors:** Madison A. Smith, Jesica Gonzalez, Anjum Hussain, Rachel N. Oldfield, Kathryn A. Johnston, Karlo M. Lopez

**Affiliations:** ^1^MI-SWACO, Shafter, CA 93263, USA; ^2^University of California, San Francisco, San Francisco, CA 94143, USA; ^3^California State University, Bakersfield, Bakersfield, CA 93311, USA

## Abstract

Lysyl oxidase is an important extracellular matrix enzyme that has not been fully characterized due to its low solubility. In order to circumvent the low solubility of this enzyme, three solubility tags (Nus-A, Thioredoxin (Trx), and Glutathione-S-Transferase (GST)) were engineered on the N-terminus of mature lysyl oxidase. Total enzyme yields were determined to be 1.5 mg for the Nus-A tagged enzyme (0.75 mg/L of media), 7.84 mg for the Trx tagged enzyme (3.92 mg/L of media), and 9.33 mg for the GST tagged enzyme (4.67 mg/L of media). Enzymatic activity was calculated to be 0.11 U/mg for the Nus-A tagged enzyme and 0.032 U/mg for the Trx tagged enzyme, and no enzymatic activity was detected for the GST tagged enzyme. All three solubility-tagged forms of the enzyme incorporated copper; however, the GST tagged enzyme appears to bind adventitious copper with greater affinity than the other two forms. The catalytic cofactor, lysyl tyrosyl quinone (LTQ), was determined to be 92% for the Nus-A and Trx tagged lysyl oxidase using the previously reported extinction coefficient of 15.4 mM^−1 ^cm^−1^. No LTQ was detected for the GST tagged lysyl oxidase. Given these data, it appears that Nus-A is the most suitable tag for obtaining soluble and active recombinant lysyl oxidase from* E. coli* culture.

## 1. Introduction

Lysyl oxidase (LOX) is a copper-dependent amine oxidase that catalyzes a key cross-linking step in collagen and elastin [1, 2]. The resulting network of cross-linked molecules serves to provide flexibility and stability within connective tissues in the cardiovascular, respiratory, and skeletal systems of biological organisms [1, 3]. Aside from its structural role in the extracellular matrix, LOX has also been implicated as playing a role in other biological processes including morphogenesis and repair of connective tissues, developmental control, and chemotaxis [2, 4, 5].

**Figure 1:**
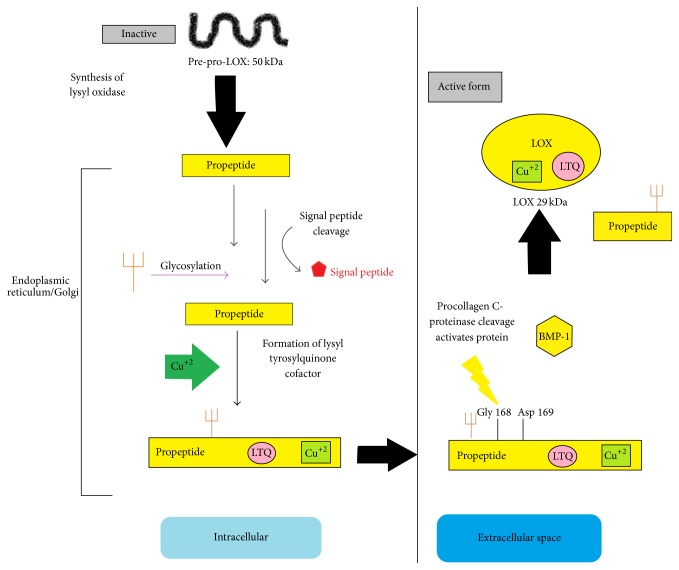
Schematic of the synthesis of active lysyl oxidase. An inactive propeptide is glycosylated and exported to the extracellular matrix following the incorporation of copper and the formation of the LTQ catalytic cofactor. The propeptide region is cleaved by procollagen C-proteinase at Gly 168 and Asp 169, yielding the propeptide and the 29 kDa active mature enzyme.

LOX is synthesized as an N-glycosylated 50 kDa propeptide [6] that is exported to the extracellular matrix where it undergoes proteolytic cleavage by procollagen-C-proteinase (bone morphogenetic protein I) at a specific gly-asp bond, confirmed to be Gly 168 and Asp 169 in humans [7, 8]. LOX is dependent on two cofactors: a tightly bound Cu(II) atom [9], which is required for the posttranslational modification of Tyr 355 to form the second cofactor, lysyl tyrosyl quinone (LTQ) [10, 11]. LTQ is formed by the cross-linking of Tyr 355 with Lys 320 to form the catalytic site of the enzyme.  Figure 1 is a schematic of the synthesis of LOX both in the intracellular space (left panel) and the extracellular matrix (right panel). It has been postulated that the copper atom is required for the catalytic activity of the enzyme [1, 9]; however, this postulate has been disputed [12].

Until recently, it has been extremely difficult to obtain large amounts of mammalian recombinant LOX due to the low solubility of the enzyme in aqueous buffers. Previous work with overexpression systems required that the enzyme be refolded [13, 14], a process that required lengthy dialysis in several different buffers. A breakthrough was achieved in 2010 when an overexpression system was developed that allowed for the production of recombinant lysyl oxidase which was purified, in active form, directly from* E. coli* culture [15]. Although this system removed the need for a lengthy refolding procedure and thereby greatly advanced the way by which this enzyme could be purified, it relied on the use of 6 M urea in the purification buffers in order to maintain the enzyme in solution.

The main focus of this work was to develop a purification system whereby the enzyme could be purified directly from* E. coli* but no longer need 6 M urea in the purification buffer. Although the yields and activity of the solubility-tagged enzyme are lower than those our laboratory reported in 2010 [15], the ability to produce an active enzyme without the need for high concentration urea is a significant advancement in the study of mammalian lysyl oxidase. These chimeric enzymes can be used to evaluate mechanical function in engineered ligaments [16] or to generate hydrogels for* in situ* bone regeneration which requires the cross-linking of lysine residues [17] among many other uses. Herein, we present the study of three different solubility tags, Nus-A, Thioredoxin (Trx), and Glutathione-S-Transferase (GST), and report their effectiveness in the isolation of soluble (nonurea) and active lysyl oxidase. The data indicates that the Nus-A solubility tag is the most effective, yielding 1.5 mg of soluble and active LOX (0.11 U/mg).

## 2. Materials and Methods

All PCR materials were obtained from New England Biolabs (NEB) with the exception of the primers which were purchased from Midland Certified Reagent Company and the polymerase which was purchased from Agilent. Unless otherwise stated, all materials were purchased from VWR International, LLC, or Research Products International Corporation.

### 2.1. Generation of pLOX06 (Trx), pLOX09 (Nus-A), and pLOX14 (GST) Constructs

The LOX gene was isolated from plasmid pLOX02 by PCR using the primer sets shown in Table 1. These primer sets introduced the corresponding restriction sites which are also listed in Table 1.

The pET vector system was used to introduce the solubility tags. PCR products and the corresponding vectors were digested with the appropriate restriction enzymes and ligated using NEB's quick ligase kit. pET32b was used to introduce the Trx solubility tag. The insertion points were at the MscI (5′-) and XhoI (3′-) restriction sites. pET42b was used to introduce the GST solubility tag. The insertion points were at the HindIII (5′-) and XhoI (3′-) restriction sites. pET43.1b was used to introduce the Nus-A solubility tag. The insertion points were at the SmaI (5′-) and XhoI (3′-) restriction sites. The ligation reactions were then transformed into NEB 10*β* competent* E. coli* cells and the resulting plasmids were isolated using a QIAGEN plasmid isolation kit in sufficient amounts for sequencing and transformation into an expression cell line.

### 2.2. Expression and Purification of Tagged Lysyl Oxidase

Three different sets of NEB SHuffle competent* E. coli* cells each independently containing the pLOX06, pLOX09, and pLOX14 plasmids were plated on LB/ampicillin plates (pLOX06 and pLOX09) or LB/kanamycin (pLOX14) and were incubated at 37°C overnight. Ampicillin concentration was set to 100 *μ*g/mL and kanamycin concentration was set to 50 *μ*g/mL. A single colony was grown overnight in 50 mL of LB media, at 37°C, containing the appropriate concentration of antibiotic. Four Erlenmeyer flasks, each containing 500 mL of Terrific Broth and the appropriate concentration of antibiotic, were inoculated with 10 mL of overnight culture. These cultures were grown, at 37°C, to an O.D._600_ of 0.8 and induced with 2 mM IPTG. Following induction, the heat was turned off and the cells were further incubated at room temperature overnight. Cells were harvested by centrifugation at 4300 ×g for 15 min in a Beckman Coulter Allegra X-14R centrifuge using a SX4750 swinging bucket rotor. Following centrifugation, cells were frozen at −80°C and lysed by sonication. The lysis/wash buffer was made up of 50 mM Tris-Cl, pH 7.6, 200 mM NaCl, and 20 mM imidazole. The cell lysate was then centrifuged at 4300 ×g. Following centrifugation, the supernatant was passed through a Ni-NTA gravity column in a 4°C cold room. The column was then washed with 200 mL of lysis/wash buffer to remove any residual cellular proteins. The tagged LOX was eluted from the column with elution buffer made up of 50 mM Tris-Cl, pH 7.6, 200 mM NaCl, and 250 mM imidazole. The isolated and purified enzyme was visualized by SDS-PAGE and the correct molecular weight was verified by using molecular weight markers. In order to ensure that the enzyme's active site was loaded with copper, the purified enzyme was dialyzed against 50 mM Tris-Cl, pH 7.6, 200 mM NaCl, and 1 mM CuSO_4_ overnight, followed by extensive dialysis against 50 mM Tris-Cl, pH 7.6, 200 mM NaCl, and 2 mM EDTA. This is a modified protocol based on previous studies addressing copper dialysis of lysyl oxidase [18, 19]. EDTA dialysis was carried out for at least three days with buffers being exchanged at a minimum twice per day. Finally, the enzyme was dialyzed against 50 mM Tris-Cl, pH 7.6, and 200 mM NaCl to remove the EDTA.

### 2.3. Characterization of LOX Activity

Properly folded LOX samples were prepared in 50 mM Tris-Cl, pH 7.6, and 200 mM NaCl and the following amounts of enzyme were used in activity assays: Trx tagged LOX 76 *μ*g, Nus-A tagged LOX 84 *μ*g, and GST tagged LOX 140 *μ*g. Coupled fluorescence activity assays for H_2_O_2_ were carried out at 37°C with 1,5-diaminopentane as the substrate [20]. Substrate concentration was 10 mM per assay. Inhibition of enzyme activity was attempted by adding *β*-APN during the assay. Assays were run in quadruplicate and specific activity is reported as a mean of all assays. Specific activity was determined by standardization using H_2_O_2_, itself standardized by titration with permanganate, and is reported in *μ*mol H_2_O_2_ produced per minute per mg of enzyme under the conditions of the assay. Thus this specific activity is not directly comparable to activities reported using different assays or different assay conditions.

### 2.4. Copper Quantification

A bicinchoninic acid assay was used to determine copper content [15]. 250 *μ*L of a 300 mg/mL trichloroacetic acid solution was added to 750 *μ*L of a 2x dilution of LOX enzyme. The resulting cloudy solution was centrifuged at 13,400 rpm for 5 min to remove precipitated enzyme. 500 *μ*L of the supernatant was combined with 100 *μ*L of a 0.34 mg/mL ascorbate solution and 400 *μ*L of HEPES/BCA solution (1.8 g NaOH, 7.8 g HEPES, 3 mL of a 6% BCA reagent A solution (Pierce), and 45 mL ddH_2_O). The absorbance of the resulting solution was measured at 362 nm on a Shimadzu UVmini-1240 spectrophotometer. A standard curve was generated by replacing the enzyme with increasing concentrations of copper from 0 to 58 *μ*M in the assay.

### 2.5. Phenylhydrazine Inhibition

Excess phenylhydrazine was added to a 1 mL solution of LOX and the solution was incubated at room temperature in the dark (microcentrifuge tube was covered with aluminum foil and stored in a drawer) overnight. The following day the solution was scanned from 800 nm to 200 nm on a Shimadzu UV-2401PC spectrophotometer.

## 3. Results

**Figure 2:**
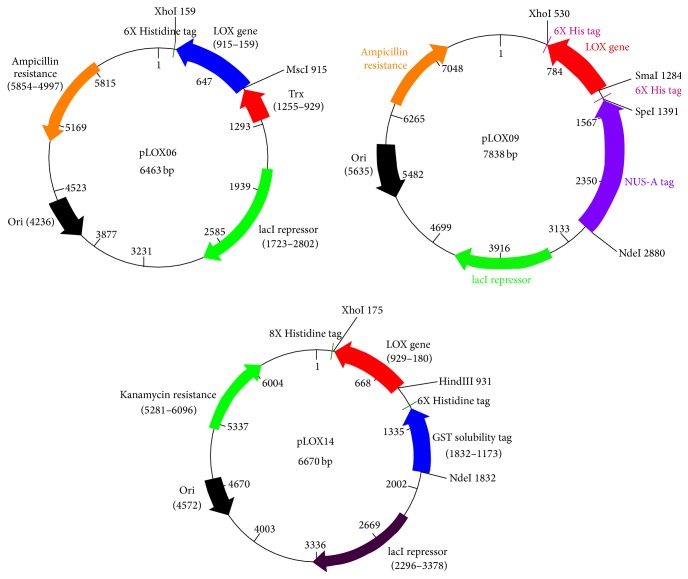
Plasmid maps for pLOX06 (Trx), pLOX09 (Nus-A), and pLOX14 (GST). The maps indicate the direction of transcription, the location of the solubility tags, and the insertion points for the wild-type mature LOX gene.

The DNA cassette corresponding to the mature form of lysyl oxidase, starting at Asp 169, was successfully isolated from plasmid pLOX02 using PCR. This cassette was inserted into pET32b, pET42b, and pET43.1b to generate plasmids pLOX06, pLOX14, and pLOX09, respectively. The plasmid maps for each of these constructs are shown in Figure 2.

**Figure 3:**
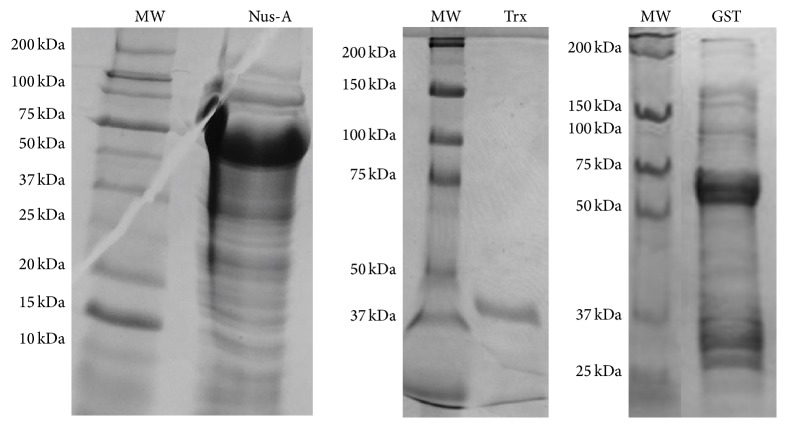
SDS-PAGE gel slices showing overexpressed, solubility-tagged lysyl oxidase. The molecular weight of each solubility-tagged enzyme was determined using the ladder on the left.

Expression was under the control of the T7 promoter in all vectors. Upon induction with 2 mM IPTG at 37°C, LOX was successfully expressed with yields of 1.5 mg for the Nus-A tagged enzyme (0.75 mg/L of media), 7.84 mg for the Trx tagged enzyme (3.93 mg/L media), and 9.33 mg for the GST tagged enzyme (4.67 mg/L of media). Purity was verified by SDS-PAGE and is shown in Figure 3. The calculated molecular weight for each construct was determined using the online bioinformatics program, ProtParam. For the pLOX09 construct (Nus-A) the calculated MW was 89023 Da, for the pLOX06 construct (Trx) the calculated MW was 42,359 Da, and for the pLOX14 construct (GST) the calculated molecular weight was 64380 Da.

**Figure 4:**
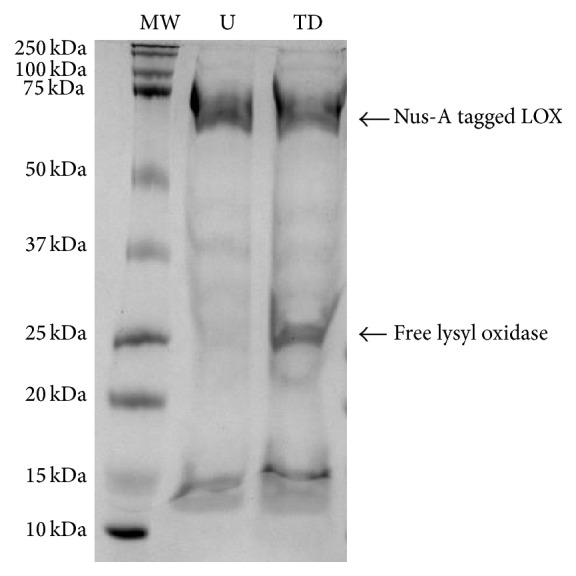
Thrombin digest of Nus-A tagged lysyl oxidase. Lane 1: molecular weight marker (MW); Lane 2: undigested tagged enzyme (U); Lane 3: thrombin digested Nus-A tagged LOX (TD).

In order to ascertain the effect of the enzyme on the solubility of LOX, a thrombin cleavage site was engineered between the Nus-A tag and the LOX enzyme. The enzyme was cleaved overnight with thrombin and imaged on 12% SDS-PAGE shown in Figure 4.

Following digestion, the purification of the free LOX enzyme was attempted; however, once the solubility tag was removed, the enzyme crashed out of solution since no urea was used in the buffer.

Following purification, the tagged LOX was assayed for enzymatic activity using a peroxide-coupled assay and 1,5-diaminopentane as the substrate. One unit of amine oxidase activity is defined as the activity resulting in oxidation of 1 *μ*mol of 1,5-diaminopentane per minute at 37°C. The conversion of 1,5-diaminopentane to the corresponding aldehyde was monitored by measuring the increase in fluorescence (Table 2).

This increase is due to the appearance of the Amplex Red product, resorufin. The activity for LOX was found to be 0.11 U/mg for the Nus-A tagged enzyme and 0.032 U/mg for the Trx tagged enzyme, and no detectable activity was observed for the GST tagged enzyme. The activity of the Nus-A tagged enzyme is consistent with the previously reported activity of 0.097 U/mg by Jung et al. [13], although only one-third as active as the previously reported value by our laboratory of the untagged but urea-dependent enzyme [15]. The activity of the Trx enzyme is much lower than that observed by Jung et al. or our laboratory, yet we consider it “active” in that this value is still three times higher than those observed in the initial experiment that identified LTQ as the catalytic cofactor [11]. For the GST tagged enzyme no activity was detected.

Normally, rapid and complete inhibition of* native* LOX is achieved by low levels (0.5 mM) of *β*-APN [21]. For the two* recombinant* constructs showing activity, inhibition with *β*-APN was achieved with high concentrations (>2.5 mM) and only after long incubation periods. This behavior has been observed for recombinant LOX in previous experiments by our laboratory as well as others [15].

This enzyme is dependent on Cu(II) for the formation of LTQ and, potentially for activity, the amount of copper present in the enzyme was determined and is presented herein as a percent incorporation. The percentage values represent moles of copper present following dialysis against EDTA per mole of purified LOX enzyme. Copper concentration was determined to be 1.70 *μ*M corresponding to 68.4% copper incorporation for the Nus-A tagged enzyme and 3.14 *μ*M corresponding to 74% copper incorporation for the Trx tagged enzyme. Although the GST tagged lysyl oxidase was dialyzed extensively against EDTA, it appears that this form of the enzyme binds adventitious copper much tighter as copper incorporation was determined to be 200%. In order to ascertain that the solubility tags were not incorporating copper, the empty pET32b, pET42b, and pET43.1b were used to overexpress the Trx, GST, and Nus-A tags, respectively. These tags were treated in the same fashion as the tagged enzyme and assayed in triplicate for copper content. The Trx tag showed 0.70% copper incorporation, the Nus-A tagged had no detectable copper incorporation, and the GST tagged showed 3% copper incorporation.

**Figure 5:**
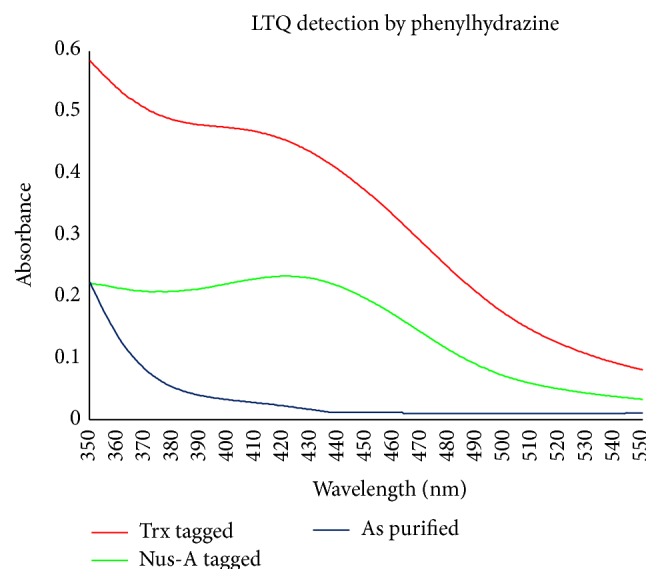
Phenylhydrazone adduct that is formed when LTQ reacts with phenylhydrazine. Red curve shows the adduct for the Trx tagged enzyme while the green curve shows the adduct for the Nus-A enzyme. The blue trace is that of LOX as purified prior to reacting with phenylhydrazine.

The presence of the LTQ cofactor was verified by monitoring enzyme inhibition by derivatization with phenylhydrazine. LOX samples at a concentration of 0.18 mg/mL for the Trx tagged enzyme and 0.64 mg/mL for the Nus-A tagged enzyme were incubated with excess phenylhydrazine at room temperature overnight in the dark. Scans were then taken the following day and the appearance of the phenylhydrazone adduct was detected and is shown in Figure 5. Using the established extinction coefficient of 15.4 mM^−1 ^cm^−1^ [10], 92% of the LTQ present in the copper-loaded LOX enzyme was titratable with phenylhydrazine.

## 4. Discussion

The isolation of active lysyl oxidase in large quantities from an overexpression system has been elusive. Attempts have been made to isolate soluble enzyme from Chinese hamster ovary cells [22] which yielded very little enzyme, as well as using* E. coli* [13, 15] which required long refolding procedures and/or the use of 6 M urea in order to solubilize the enzyme.

This work focused on elucidating a method to obtain milligram quantity yields of purified* recombinant* LOX without using urea, in any fashion, in the purification buffers. The data presented here shows that this can be successfully accomplished using an* E. coli* system and nutrient rich media if a solubility tag is tethered to LOX. In particular, the data indicates that the most suitable solubility tag is Nus-A because it yields milligram quantities of the enzyme that is soluble without needing urea, has high activity, and incorporates copper, and the LTQ cofactor is present. Given that both GST and Trx tagged LOX yielded higher amounts of LOX, it appears that the smaller tags interfere less with the overexpression levels; however, the presence of smaller tags appears to inhibit the enzyme to a large degree. In the case of the GST tagged lysyl oxidase, it is postulated that the GST tag actually produces a misfold in the enzyme that causes it not only to be inactive but also to bind adventitious copper with high affinity. The copper incorporation of the chimeric enzymes does not appear to be linked to the solubility tags as control experiments show that the tags, in and of themselves, do not incorporate copper.

The presence of the LTQ cofactor, verified and quantified by the formation of a phenylhydrazone adduct, indicates that the Trx and Nus-A solubility tags do not interfere with the formation of LTQ. This is important because the primary function of the tag is to keep the enzyme soluble; hence, it will not be cleaved when carrying out studies on LOX. If the tag is cleaved, the added solubility is removed and the enzyme will crash out of solution.

Future studies on the use of solubility tags with LOX include a comparison of the tagged enzyme with the lysyl oxidase-like isoforms of the enzyme which, unlike LOX, are much more soluble and which retain their N-terminal peptide region.

Lastly, the ability to harvest active LOX in large quantities, now fully soluble in aqueous buffer, puts us one step closer to the ultimate goal with this enzyme: the elucidation of its crystal structure. The tags used in the study were selected specifically because they have been used in previous work to crystallize insoluble proteins without the need to cleave them off [23]. To date the only amine oxidase crystal structures have been of copper-containing amine oxidase enzymes of the TPQ-containing EC1.4.3.6 class, not the EC1.4.3.13 class of LTQ-containing lysyl oxidases. This endeavor has eluded us for far too long and our laboratory is currently working on screening these tagged enzymes in an effort to identify conditions suitable for growing crystals.

## Figures and Tables

**Table 1:** Listing of primers used for isolating the LOX gene. Each primer introduced a different restriction site at the 5′-end in order to insert the resulting PCR product into the corresponding vector in the correct orientation. The reverse primer (MLOXR) was the same for all PCR reactions.

Primer name	Primer sequence	Restriction site
Trx tag (pLOX06 forward primer)	5′-AAGGGG**TGGCCA**GGACGACCCTTACAACCCCTAC-3′	MscI at 5′-end
LOX tag (pLOX09 forward primer)	5′-AAGGGG**CCCGGG**ACGACCCTTACAACCCC-3′	SmaI at 5′-end
MLOXGSTF (pLOX14 forward primer)	5′-AAGGGG**AAGCTT**ATGGACGACCCTTACAACCCC-3′	HindIII at 5′-end
MLOXR (reverse primer for all constructs)	5′-AAGGGG**CTCGAG**ATACGGTGAAATTGTGCAGCC-3′	XhoI at 3′-end

**Table 2:** Yield, activity, and copper incorporation of each tagged form of lysyl oxidase. The line labeled LOX is data from a previously published paper [15] related to untagged and urea solubilized LOX. This enzyme was not subjected to copper dialysis and hence the lower copper incorporation.

	Protein yield (mg)	Specific activity (U/mg)	Copper incorporation
LOX	10.0	0.31	19%
LOX Nus-A tag	1.5	0.11	68%
LOX Trx tag	7.8	0.03	74%
LOX GST tag	9.3	None Detected	270%

One activity unit is defined as 1 *μ*mol of H_2_O_2_ produced per minute per mg of enzyme at 37°C.

## Abbreviations

### Abbreviations

LOX: Lysyl oxidase
LTQ: Lysyl tyrosyl quinone
IPTG: Isopropyl-*β*-D-thiogalactopyranoside
Trx: Thioredoxin
GST: Glutathione-S-Transferase.

## References

[1] Kagan H. M., Trackman P. C. (1991). Properties and function of lysyl oxidase. *American Journal of Respiratory Cell and Molecular Biology*.

[2] Lucero H. A., Kagan H. M. (2006). Lysyl oxidase: an oxidative enzyme and effector of cell function. *Cellular and Molecular Life Sciences*.

[3] Kagan H. M., Li W. (2003). Lysyl oxidase: properties, specificity, and biological roles inside and outside of the cell. *Journal of Cellular Biochemistry*.

[4] Csiszar K. (2001). Lysyl oxidases: a novel multifunctional amine oxidase family. *Progress in Nucleic Acid Research and Molecular Biology*.

[5] Li W., Liu G., Chou I.-N., Kagan H. M. (2000). Hydrogen peroxide-mediated, lysyl oxidase-dependent chemotaxis of vascular smooth muscle cells. *Journal of Cellular Biochemistry*.

[6] Trackman P. C., Bedell-Hogan D., Tang J., Kagan H. M. (1992). Post-translational glycosylation and proteolytic processing of a lysyl oxidase precursor. *The Journal of Biological Chemistry*.

[7] Panchenko M. V., Stetler-Stevenson W. G., Trubetskoy O. V., Gacheru S. N., Kagan H. M. (1996). Metalloproteinase activity secreted by fibrogenic cells in the processing of prolysyl oxidase. Potential role of procollagen C-proteinase. *The Journal of Biological Chemistry*.

[8] Uzel M. I., Scott I. C., Babakhanlou-Chase H. (2001). Multiple bone morphogenetic protein 1-related mammalian metalloproteinases process pro-lysyl oxidase at the correct physiological site and control lysyl oxidase activation in mouse embryo fibroblast cultures. *The Journal of Biological Chemistry*.

[9] Gacheru S. N., Trackman P. C., Shah M. A. (1990). Structural and catalytic properties of copper in lysyl oxidase. *The Journal of Biological Chemistry*.

[10] Bollinger J. A., Brown D. E., Dooley D. M. (2005). The formation of lysine tyrosylquinone (LTQ) is a self-processing reaction. Expression and characterization of a *Drosophila lysyl* oxidase. *Biochemistry*.

[11] Wang S. X., Mure M., Medzihradszky K. F. (1996). A crosslinked cofactor in lysyl oxidase: redox function for amino acid side chains. *Science*.

[12] Mure M., Mills S. A., Klinman J. P. (2002). Catalytic mechanism of the topa quinone containing copper amine oxidases. *Biochemistry*.

[13] Jung S. T., Kim M. S., Seo J. Y., Kim H. C., Kim Y. (2003). Purification of enzymatically active human lysyl oxidase and lysyl oxidase-like protein from *Escherichia coli* inclusion bodies. *Protein Expression and Purification*.

[14] Ouzzine M., Boyd A., Hulmes D. J. (1996). Expression of active, human lysyl oxidase in *Escherichia coli*. *FEBS Letters*.

[15] Herwald S. E., Greenaway F. T., Lopez K. M. (2010). Purification of high yields of catalytically active lysyl oxidase directly from *Escherichia coli* cell culture. *Protein Expression and Purification*.

[16] Lee C. A., Lee-Barthel A., Marquino L., Sandoval N., Marcotte G. R., Baar K. (2015). Estrogen inhibits lysyl oxidase and decreases mechanical function in engineered ligaments. *Journal of Applied Physiology*.

[17] Sánchez-Ferrero A., Mata Á., Mateos-Timoneda M. A. (2015). Development of tailored and self-mineralizing citric acid-crosslinked hydrogels for in situ bone regeneration. *Biomaterials*.

[18] Kim M. S., Kim S.-S., Jung S. T. (2003). Expression and purification of enzymatically active forms of the human lysyl oxidase-like protein 4. *The Journal of Biological Chemistry*.

[19] Lopez K. M., Greenaway F. T. (2011). Identification of the copper-binding ligands of lysyl oxidase. *Journal of Neural Transmission*.

[20] Palamakumbura A. H., Trackman P. C. (2002). A fluorometric assay for detection of lysyl oxidase enzyme activity in biological samples. *Analytical Biochemistry*.

[21] Tang S. S., Trackman P. C., Kagan H. M. (1983). Reaction of aortic lysyl oxidase with *β*-aminopropionitrile. *The Journal of Biological Chemistry*.

[22] Kagan H. M., Reddy V. B., Panchenko M. V. (1995). Expression of lysyl oxidase from cDNA constructs in mammalian cells: the propeptide region is not essential to the folding and secretion of the functional enzyme. *Journal of Cellular Biochemistry*.

[23] Smyth D. R., Mrozkiewicz M. K., McGrath W. J., Listwan P., Kobe B. (2003). Crystal structures of fusion proteins with large-affinity tags. *Protein Science*.

